# Electromechanical Coupling in Collagen Measured under Increasing Relative Humidity †

**DOI:** 10.3390/ma16176034

**Published:** 2023-09-02

**Authors:** Arwa Bazaid, Fengyuan Zhang, Qiancheng Zhang, Sabine Neumayer, Denise Denning, Stefan Habelitz, Ana Marina Ferreira, Brian J. Rodriguez

**Affiliations:** 1 School of Physics and Conway Institute of Biomolecular and Biomedical Research, University College Dublin, Dublin D04 V1W8, Ireland; arwabazaid@gmail.com (A.B.); zhangfy@mail.sustech.edu.cn (F.Z.); qiancheng.zhang@ucd.ie (Q.Z.); neumayersm@ornl.gov (S.N.); 2 FOCAS Research Institute, Technological University Dublin, City Campus, Camden Row, Dublin D04 V1W8, Ireland; denise.denning@tudublin.ie; 3 Department of Preventative and Restorative Dental Sciences, School of Dentistry, University of California, San Francisco, CA 94143, USA; stefan.habelitz@ucsf.edu; 4 School of Engineering, Newcastle University, Newcastle upon Tyne NE1 7RU, UK; ana.ferreira-duarte@newcastle.ac.uk

**Keywords:** collagen, piezoelectricity, atomic force microscopy, piezoresponse force microscopy, mechanotransduction, humidity

## Abstract

The functional role of collagen piezoelectricity has been under debate since the discovery of piezoelectricity in bone in 1957. The possibility that piezoelectricity plays a role in bone remodeling has generated interest in the investigation of this effect in relevant physiological conditions; however, there are conflicting reports as to whether collagen is piezoelectric in a humid environment. In macroscale measurements, the piezoelectricity in hydrated tendon has been shown to be insignificant compared to dehydrated tendon, whereas, at the nanoscale, the piezoelectric effect has been observed in both dry and wet bone using piezoresponse force microscopy (PFM). In this work, the electromechanical properties of type I collagen from a rat tail tendon have been investigated at the nanoscale as a function of humidity using lateral PFM (LPFM) for the first time. The relative humidity (RH) was varied from 10% to 70%, allowing the piezoelectric behavior to be studied dry, humid, as well as in the hydrated range for collagen in physiological bone (12% moisture content, corresponding to 40–50% RH). The results show that collagen piezoresponse can be measured across the humidity range studied, suggesting that piezoelectricity remains a property of collagen at a biologically relevant humidity.

## 1. Introduction

Since the discovery of piezoelectricity in bone in 1957 [1], the piezoelectric properties of collagen have been hypothesized as the primary mechanism of strain-generated potentials in dry bone; however, streaming potential [2] and flexoelectricity [3] have also been implicated in bone electromechanics. Furthermore, it has been reported that dry bone and tendons hold piezoelectric properties in their own right. While early studies concluded that the generated electrical potential in fully hydrated tendon is from streaming potentials [4], this was reported to be almost zero with 45% moisture content in wet bone [1]. In a subsequent work, Anderson et al. measured the piezoelectric properties of wet bone in solutions of varying pH. They reported that the piezoelectric constant varied with pH, indicating the contribution of streaming potential to the measured response [5]. Additionally, some studies attributed the loss of piezoelectricity in collagen, under wet conditions, to a change in the structure of collagen when bound to water. They suggested the bone-water interactions increased the symmetry the of collagen molecules, precluding piezoelectric properties [4,5]. Pienkowski and Pollack have reported a reduction in the magnitude of generated electric potentials in wet bone with increasing solution conductivity and viscosity, yielding results consistent with a streaming potential mechanism. These results lead to the role of piezoelectricity in bone remodeling being widely discarded in subsequent literature [5,6,7,8].

Conflicting results supporting the existence of piezoelectricity in wet bone were first reported by Basset and Becker in 1962 [9]. Subsequent reports indicated that piezoelectricity still manifested itself in fully hydrated bone and tendon [5,9,10,11]. A review by Ahn and Grodzinsky proposed that the piezoelectric charge generated from applied mechanical stress increased the magnitude of the zeta potential and consequently contributed to higher streaming potentials, thereby demonstrating that both piezoelectricity and streaming potential contributed to strain-generated potentials [12].

Significant efforts have been made toward understanding the role piezoelectricity plays in mechanotransduction. It is essential, however, to understand the electromechanical coupling down to the nanoscale, since there are several macroscopic studies doubting that collagen could behave as a piezoelectrical material when fully hydrated. A study by Halperin et al. has confirmed the piezoelectric properties of dry and wet bone using piezoresponse force microscopy (PFM) [13]. No difference was observed in the measured piezoelectric deformation from transverse cuts of wet and dry bone samples, recorded as the cantilever oscillation resulting from applying bias to the conductive tip. In order to further understand the effect of mechanical loading on bone remodeling, an experiment was devised to investigate piezoelectricity in collagen as a function of humidity, whereby humidity was ramped above the level where collagen saturates 70% relative humidity (RH) [14]. Here, using lateral PFM (LPFM), we report the shear piezoelectric properties in dry and moist conditions for both isolated collagen fibrils and rat tail tendon. LPFM allowed both to quantify the electromechanical properties with nanometer-scale resolution in collagen to reveal the polar ordering within the tissues. The results show conclusively that collagen exhibits piezoelectric behavior up to 70% RH, demonstrating that piezoelectricity is a functional property of collagen in the humidity range where it would be physiologically relevant.

## 2. Methods and Materials

### 2.1. Preparation of Collagen Fibrils

Insoluble type I collagen extracted from bovine Achilles tendon (Sigma-Aldrich, Darmstadt, Germany) was swollen in 0.01 M hydrochloric acid overnight at 0 °C. The resulting solution was homogenized for ~10 min at 0 °C using a blender (MR 4000 HC, Braun, Waiblingen, Germany) and diluted in phosphate buffered saline (PBS) solution to a final concentration of 100 µg/mL. Approximately 150 µL of diluted collagen solution was deposited on a freshly cleaved mica substrate and incubated for 10 min. Subsequently, the substrate was washed with PBS (1×) and deionized water (3×) and finally dried under ambient conditions or by using compressed nitrogen [15].

### 2.2. Preparation of Rat Tail Tendon

Collagen fibril bundles were extracted from the rat tail tendon immediately after thawing to prevent degradation. The extraction process was followed as previously described by Rajan et al. [16]. Briefly, an incision was made along the length of the tail using a surgical scalpel. After removing the skin, the fibril bundles start to come away from the tendon. The bundles were rinsed with deionized water and stored at 4 °C in a PBS solution until used. Thereafter, a small section of the extracted fibril bundle (≈1 cm in length) was taken and thoroughly washed with deionized water to remove any PBS, to prevent crystallization when the sample is dried. Subsequently, the sample was placed on the surface of freshly cleaved mica and pulled, but not stretched, across the mica in the direction of the length of the bundle to keep the natural alignment of the fibrils.

### 2.3. AFM and LPFM Experiments

All imaging was performed using an atomic force microscope (AFM) (MFP-3D, Asylum Research) in contact mode at room temperature, which is maintained at approximately 20 °C. Conductive Pt-coated AFM tips of 15 kHz resonance frequency and 0.2 N/m nominal spring constant (MikroMasch, DPE-XSC11, Tallinn, Estonia) were used to image the collagen fibrils and Pt/Ir-coated tips (Nanosensors, PPP-EFM, Neuchatel, Switzerland) with a nominal resonant frequency and spring constant of 75 kHz and 2.8 N/m, respectively, were used to image tendon samples. A lower spring constant probe was required to image the individual fibrils in contact mode.

Average collagen fibril width was determined from 10 fibrils from AFM topography images. To independently observe changes in capillary forces associated with changing humidity and assess whether the application of bias modified capillary forces, adhesion between the tip and the sample as a function of increasing humidity was determined from force-distance curves. Ten points were recorded for each set of samples and all obtained force curves were analyzed using Igor Pro 6.36 (WaveMetrics, Portland, OR, USA).

LPFM was used to investigate the electromechanical properties of collagen as a function of humidity. LPFM is based on the converse piezoelectric effect where an electric field induces shear deformations of the sample, which are recorded via the torsional movement of the cantilever [17]. The local piezoelectric response was detected using an external lock-in amplifier at the first harmonic component of the cantilever torsion under the assumption that the tip follows the local bias-induced shear deformations of the sample. LPFM was performed using an AFM (MFP-3D, Asylum Research, Goleta, CA, USA) equipped with a lock-in amplifier (HF2LI, Zurich Instruments, Zürich, Switzerland). During LPFM measurements, an AC voltage (typically 30 V at 7 kHz) was applied using a high-voltage amplifier power supply (F10A, FLC Electronics AB, Boulder, CO, USA) via a conductive AFM probe. The induced deformation as a function of the applied voltage was quantified (a.u.) by single-point measurements (individual fibril; *n* = 10), (tendon; *n* = 20) and subsequently the shear piezoelectric response was determined from the slope of the resulting graph. Fibril and tendon data are normalized independently to the condition where the highest response is measured.

### 2.4. Humidity Measurements

Humidity measurements were performed using a fluid cell (Asylum Research), connected via tubing to a closed container. The container was also connected via tubing to a source of compressed nitrogen. The RH was increased by placing salt solutions of specified concentrations (Table 1) into the closed container and decreased by gentle nitrogen flow (~10%) [18]. Room humidity was ~20%, as measured with a digital hygrometer (EMR899HGN, Oregon Scientific, Portland, OR, USA) and the RH inside the fluid cell was continuously monitored using a humidity sensor (HIH-4000, Honeywell International, Inc., Charlotte, NC, USA) and a digital multimeter (7534-02, Yokogawa, Tokyo, Japan). The average RH achieved at different salt solutions are shown in Table 1. The humidity was allowed to stabilize before measurements were undertaken. Fibrils were measured first at 20%, then at 10%, 30%, 40%, and 50% RH. Tendon was measured from 10% to 70% RH in that order.

## 3. Results

### 3.1. AFM Characterization of Collagen Fibril and Rat Tail Tendon as a Function of Relative Humidity

**Figure 1:**
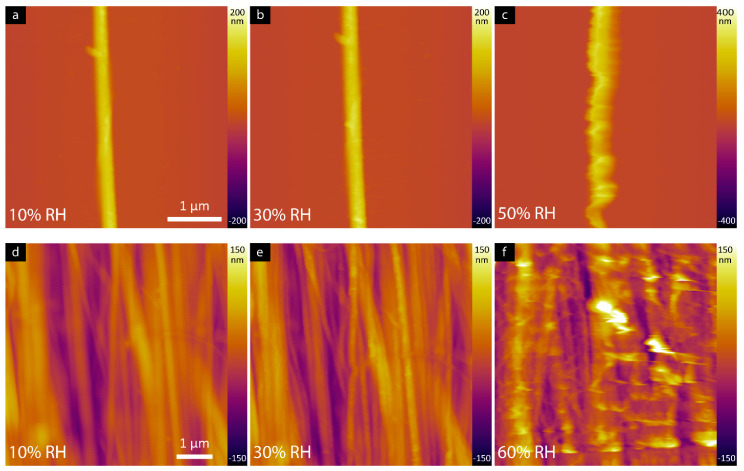
AFM topography images of individual collagen fibril (**a**–**c**) and rat tail tendon (**d**–**f**) as a function of ramping humidity.

**Figure 2:**
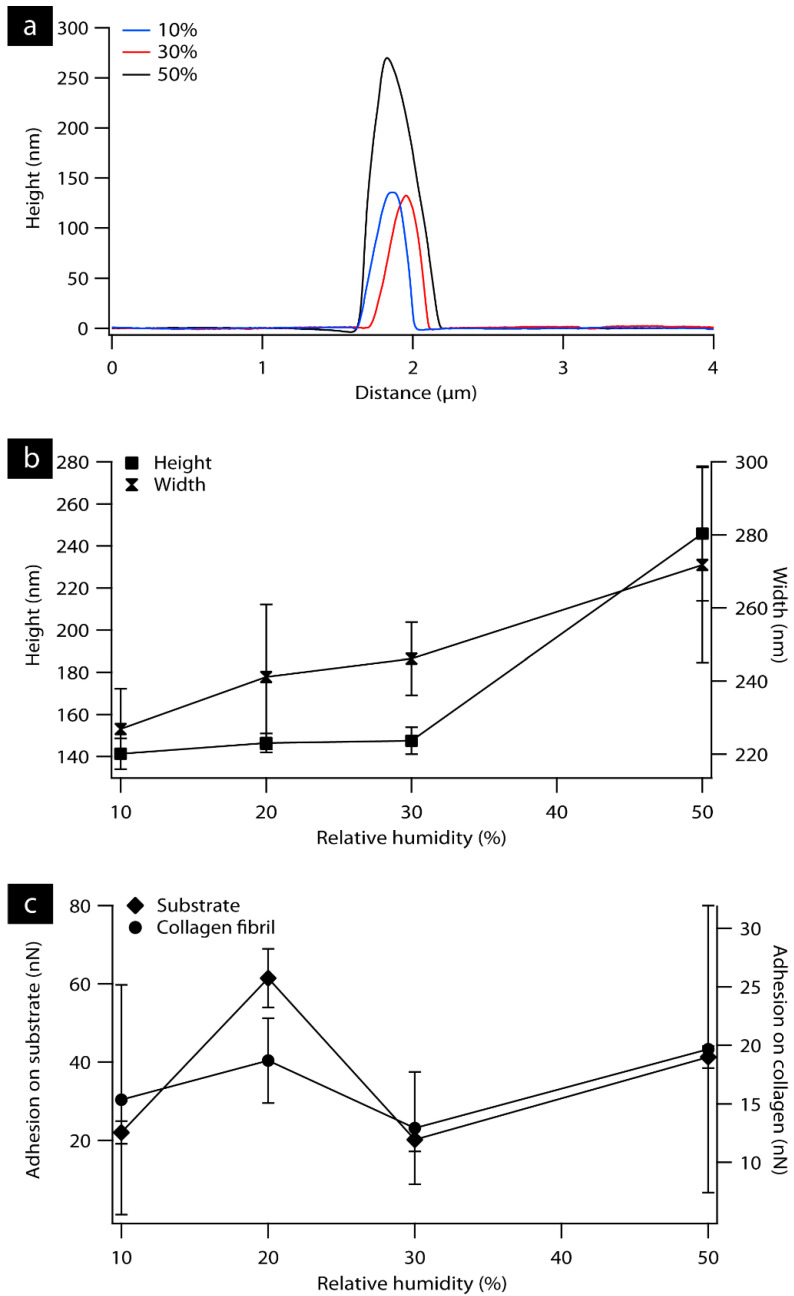
(**a**) Representative line profiles of individual collagen fibrils measured from Figure 1. (**b**) Graph displaying the effect of increasing RH on measured adhesion force on substrate and collagen fibril. (**c**) Measured height and width from AFM topography images of individual fibrils.

Surface topography was determined using atomic force microscopy (AFM) for individual collagen fibril and rat tendon samples. As RH increased, collagen fibrils appeared to swell (Figure 1a–c). In Figure 2a, representative cross-sectional line profiles of collagen fibrils (Figure 1a–c) confirm the change in the height and width of individual fibrils with increasing RH. Measurements were also performed at 60% RH, but the loading force of the tip damaged the fibril. The measured width of the fibrils increased gradually (Figure 2b) as the humidity reached 50% to 272 ± 27 nm from 227 ± 11 nm in 10% RH as measured from the AFM height images. A similar trend was observed for height measurements. Fibril height increased from 141 ± 7 nm and 148 ± 6 nm measured from 10% and 30% RH, respectively, to 246 ± 32 nm at 50% RH. The adhesion force, measured by quantifying the ‘pull-off’ force required to overcome the capillary force present between the tip and surface, are plotted as a function of humidity for the mica substrate and collagen fibril as can be shown in Figure 2c. On the mica substrate, the adhesion force increased by a factor of 3 from 22 ± 3 nN measured at 10% RH to 61 ± 7 nN at 20% RH. In contrast, the measured adhesion force on collagen fibril shows minor changes over the entire humidity range.

Similar measurements were also conducted on rat tail tendon to study the effect of increasing humidity on natural tissues. Figure 1d–f displays AFM height images of the tendon at 10%, 30%, and 60% RH, the characteristic periodic banding was revealed to be 68.8 ± 0.2 nm, 67.9 ± 0.3 nm and 67.7 ± 0.4, respectively. There are no observable changes in the measured periodicity as the tendon sample exposed to increasing RH (10–70% RH). Fibril widths and heights are not measured given the structure of the tendon. Adhesion measurements on tendon are discussed later.

### 3.2. LPFM Analysis as a Function of Relative Humidity

**Figure 3:**
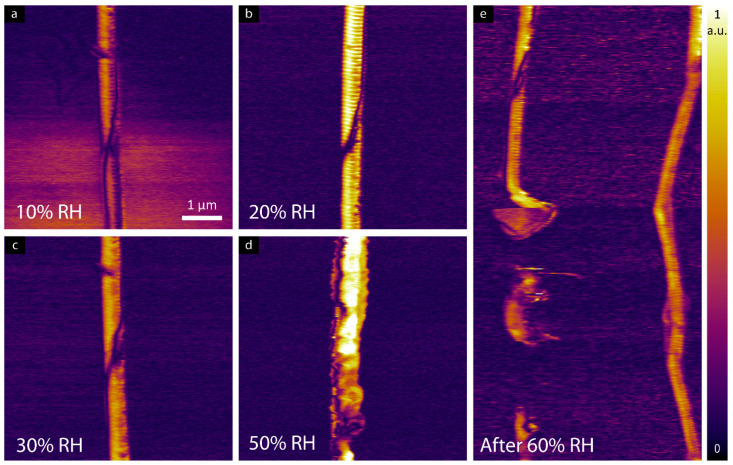
LPFM amplitude images of individual collagen fibril at (**a**) 10%, (**b**) 20%, (**c**) 30% and (**d**) 50% RH. (**e**) shows the deformation of collagen fibril while exposed to 60% RH, note that the image was recorded in ambient conditions.

To study the effect of ramping RH on the electromechanical properties of collagen, individual collagen fibrils have been studied by LPFM. The LPFM amplitude images of the collagen fibril are presented in Figure 3 wherein a higher piezoresponse signal was observed when samples were measured at 20% RH in comparison to the measured signal at 10% RH.

**Figure 4:**
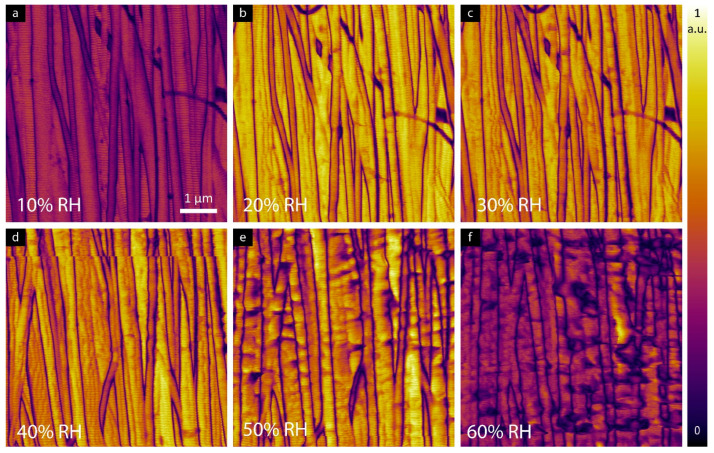
LPFM amplitude images of rat tail tendon at (**a**) 10%, (**b**) 20%, (**c**) 30%, (**d**) 40%, (**e**) 50% and (**f**) 60% RH.

**Figure 5:**
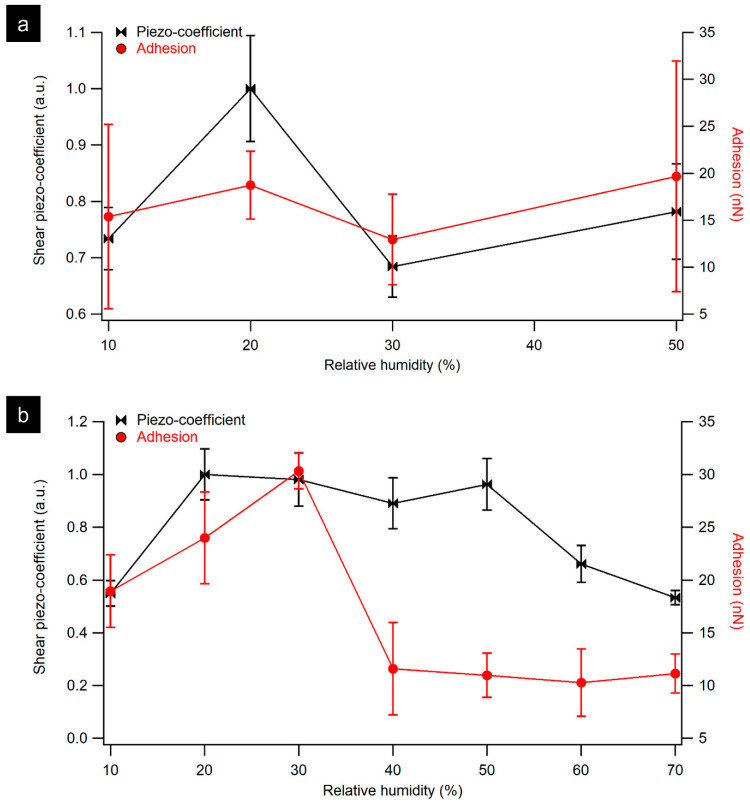
Graph displaying the measured shear piezoresponse and adhesion force on individual collagen fibril (**a**) and rat tail tendon (**b**) as a function of increasing RH.

LPFM amplitude images shown in Figure 4 confirm piezoelectricity in tendon samples up to 60% RH. At a humidity of 10%, a reduction in the magnitude of the shear piezoelectric response is observed, as shown in Figure 5a. When increasing the humidity from 20% to 50%, a higher piezoresponse signal was detected compared to the sample at 10% RH. Further increases in RH above 60% resulted in a reduction of the measured shear response.

To assess whether the application of bias necessary for LPFM modified the capillary forces, adhesion was also measured. With increasing humidity (>30% RH) a dramatic decrease in the measured adhesion force was observed, as can be seen in Figure 5b. A gradual increase was observed in adhesion force at 20% RH followed by a clear transition point at 30% as it reaches 30 ± 2 nN. It is also notable that above 30% RH, a sharp decrease was recorded in the measured force to 12 ± 4 nN, followed by a steady decrease as the humidity reached 60%. A similar trend in adhesion measurements was observed as a function of increasing RH on hydrophilic surfaces, where the adhesion first increases gradually and then drops at high humidity [19,20]. Salmeron et al. reported an increase in the adhesion force at 20% that was attributed to strong capillary forces as additional water accumulates and forms a meniscus around the contact point [19].

## 4. Discussion

The role of water on the structure of collagen has been widely investigated in literature using a variety of techniques [21,22,23,24,25]. Such studies have shown hydration can greatly impact the physical properties of collagen [26]. D-band periodicity is one of the key structural features that helps identify alteration in collagenous tissues due to age or diseases [27,28,29]. Previous work by Holl et al. showed that the D-band periodicity of collagen fibrils is not altered when fully hydrated [30]. Here, and similar to the previous results, there is no significant change in the D-band periodicity at different RH for the tendon sample with differences up to ±2 nm. In the case of individual fibrils, increase in RH leads to low image resolution at the loading forces used, and thus the D-band periodicity is difficult to resolve. Tendon is composed of tightly lateral packed fibrils and thus the full width of individual fibrils from topography images cannot be fully exposed at higher RH levels. Regardless, it is clear from Figure 1d–f that fibrils become swollen with increasing RH, consistent with previous reports, e.g., Ref. [31]. From the LPFM image (Figure 2a,b) of the same area, however, it was possible to visualize individual fibrils and therefore, the width can be measured from the defined domain boundaries between areas of higher response.

The measured pull-off forces in Figure 5 show a non-monotonic behavior, increasing with increasing humidity to 30% and decreasing gradually with further increase in RH. Similar results were reported previously for the humidity dependence of the adhesion force in relation to the hydrophilicity of the tip and the sample [19,32,33,34,35]. For instance, Jang et al. reported a maximum pull-off force of around 20% for strongly hydrophilic surfaces while the measured pull-off force was independent of the humidity using a hydrophobic tip [33]. In our measurements, the maximum pull-off force was at 20% RH for individual fibril and was at 30% for tendon sample, which is comparable to the results reported by Xu et al. and Kim et al. [19,36]. However, stronger pull-off forces were observed on tendon sample 30 ± 2 nN compared to the previous values [19,36]. This change in pull-off forces may be due to Pt tips that were used which are more hydrophilic than the Si_3_N_4_ tip used in their experiments [37]. The hydrophilicity of Pt depends strongly on the preparation protocol and surface contamination [37,38].

Luescher et al. investigated collagen hydration with X-ray diffraction and reported that the adsorption of water by the helical structure of collagen saturates at a moisture content of 26% wt (~60% RH), above which water is then adsorbed within the intermolecular space [14,39]. The presented results confirm the presence of piezoelectricity in collagen as a function of increasing humidity. At low humidity, a slight decrease in the piezoelectric response was observed at 10% RH for the tendon and fibril compared to ambient conditions. As the humidity increased, higher piezoresponse was observed from samples measured between 20–60%, with a decrease above this range. Perhaps the reduced response above 60% RH is related to the presence of intermolecular water. However, screening of the electric field resulting from the applied AC voltage in a higher humidity environment might also reduce the measured piezoresponse. Future work should correlate the piezoresponse measurements under different humidities with local mechanical, structural (e.g., Fourier-transform infrared spectroscopy), and swelling investigations to determine the underlying mechanism. Decreased intermolecular spacing, reported in the case of chemical dehydration, will result in gradual shrinkage of collagen fibrils which might affect the ability of the fibril to deform [40,41]. Perhaps the reverse is true and increased deformability with increasing humidity, up to a moisture content limit, increases deformability and electromechanical coupling. A previous study reported no difference in longitudinal piezoelectric response between wet or dry bone samples [13], providing, in addition to our reported results, strong evidence of the existence of piezoelectricity in moisture-rich collagenous tissues. Our work provides evidence for the first time on how electromechanical coupling in fibrillar collagen is influenced by the degree of humidity, showing a sustained increase in the piezoresponse when RH < 60%. Collagen piezoelectricity is of great interest in different fields ranging from physics of proteins up to tissue engineering such as biomineralization processes driven by piezoelectric phenomena [42,43].

## 5. Conclusions

Lateral piezoresponse force microscopy experiments have been implemented to study the effect of increasing RH on the electromechanical properties of collagen. The results confirm that both tendon and individual collagen fibrils exhibit shear piezoelectricity across the range of RH studied, suggesting that collagen piezoelectricity is a phenomenon of hydrated collagen, in support of previous findings [13]. Further work should attempt to understand the mechanism of the moisture-dependent response observed. PFM has been previously applied in liquid [44] and in controlled humidity chambers like in our measurements, suggesting the possibility of using this technique to further investigate electromechanics of, e.g., healthy vs. pathological, collagenous tissues in physiologically relevant environments. Such work will bridge the gap between laboratory settings and physiological contexts and contribute to a comprehensive understanding of piezoelectricity as a design element in biomimetic tissue engineering applications.

## Figures and Tables

**Table 1:** Expected RH (%) achieved using different salt solutions.

Salt Solution	Salt Concentration (g/mL)	RH (%)	
		Literature [18]	Achieved
Magnesium chloride (MgCl_2_)	0.5	33%	31%
Potassium carbonate (K_2_CO_3_)	1.1	43%	43%
Magnesium nitrate (Mg (NO_3_)_2_)	1.2	53%	54%
Sodium chloride (NaCl)	0.3	75%	60%
Potassium chloride (KCl)	0.4	84%	73%

## Data Availability

The data presented in this study are available on reasonable request from the corresponding author.
